# Cultural industry development from entrepreneurship under the background of rural revitalization strategy

**DOI:** 10.3389/fpsyg.2022.959226

**Published:** 2022-08-03

**Authors:** Jing Gao

**Affiliations:** Management College, Ocean University of China, Qingdao, China

**Keywords:** rural revitalization strategy, entrepreneurship, cultural industry competitiveness analysis, back propagation artificial neural network algorithm, data envelopment analysis model

## Abstract

The implementation of the rural revitalization strategy can effectively inherit the excellent traditional Chinese culture and facilitate the comprehensive development of the cultural industry. At present, China is promoting the transformation and upgrading of its industrial structure. The criterion for measuring the “cultural soft power” of a country or region is the competitiveness of its cultural industry. The cultural industry has grown rapidly in recent years, and the overall economic benefits of the industry have also improved, effectively alleviating the employment pressure across the country. However, there are still many problems. How to accurately measure the level of competitiveness of the regional cultural industry and enhance its competitiveness is the first problem in the development of the cultural industry. It finds out the main factors that affect the competitiveness of the cultural industry in the context of rural revitalization strategy using the relevant theories of cultural industry and industrial competitiveness. Besides, the evaluation index system of cultural industry competitiveness is constructed from the perspective of the system. The projection pursuit model and data envelopment analysis model are established based on the genetic algorithm. The model is used to carry out empirical research on the competitiveness level of cultural industry in a region, and conclusions are drawn. The average projected value of the base competitiveness in the region exceeds 0.8. The average projected value of dominant competitiveness exceeds 0.7. The average projected value of potential competitiveness exceeds 1.1. This research proposes corresponding suggestions for the problems in the current growth of the cultural industry in this region through the competitiveness of this region and the level of other areas. This study can also provide some help for the in-depth study of the Chinese cultural industry.

## Introduction

China’s countryside has always been a regional complex unit integrating the three characteristics of nature, society, and economy. It is also a carrier that provides multiple functions such as production, life, ecology, and culture for most people in the country. Now, it is interdependent and mutually reinforcing with cities and towns (Li et al., 2020). In 2017, the rural revitalization strategy was proposed in the report of the 19th National Congress of the Communist Party of China. The report emphasized that agricultural, rural, and peasant issues were fundamental issues related to the national economy and people’s livelihood, and solving the “three rural” issues must always be the top priority of the Party’s work. In short, the rise and fall of the countryside affected the country (Wang and Zhuo, 2018). In particular, the current unbalanced and insufficient development is particularly prominent in rural areas. The time to solve the problem of rural development determines the time when China is in the primary stage of socialism (Liu L. et al., 2020). China’s two centenary goals are to build a moderately prosperous society and to build a great modern socialist country in an all-around way. However, the most central problem lies in the countryside. The greatest potential and stamina for development are also in the countryside (Long et al., 2019). As a special cultural and economic form, the cultural industry should also develop together with the rural revitalization strategy and promote each other (Patil et al., 2021). The development of the cultural industry is inseparable from the main body of the industry: the enterprise. Entrepreneurship is divided into innovation, risk-taking, profit creation, and opportunity cognition as an industry, an enterprise cluster, and the soul of every enterprise (Drobyazko et al., 2019). Innovation is all about asking entrepreneurs to keep innovating while challenging a dangerous and uncertain environment. Risk-taking is to require entrepreneurs to take on the judgmental decision-making results caused by the uncertain environment and innovative behavior. The purpose of business is profit. The imbalance of the market generates profits, which determines the thickness of profits. Opportunity cognition requires entrepreneurs to be aware of and successfully seize opportunities in time (Pradhan et al., 2020).

Research on the development of cultural industry has always been a hot issue both domestically and internationally. For example, Duxbury revealed cultural and creative work in rural and remote areas through themes and trajectories of multidisciplinary and international literature. It was found that these discourses have become intertwined in policy and planning documents over the past decade through a study of cultural dynamism. The rural creative class is associated with rural innovation, the rural creative economy, and creative entrepreneurship in rural and remote areas. The results suggested an opportunity to fuse these discussions into a holistic approach to promoting cultural and creative work in rural and remote areas (Duxbury, 2021). Keane described the development of China’s creative cities from urban agglomerations to “characteristic towns,” reflecting the government’s desire to build characteristic cultural brands. It also showed how recent iterations of development work in Hangzhou and its relationship to the Internet+. Internet+ was a policy blueprint launched by the Chinese government in March 2015 to support China’s ambition to become an innovative nation. The term “entrepreneurial solutionism” described a tendency to see digital technology as a solution to China’s social and economic problems. It was also a way to promote the realization of the “Chinese Dream” of national rejuvenation. The core of the Internet+ blueprint was the slogan “Mass Entrepreneurship, Mass Innovation,” alluding to the Silicon Valley-style neoliberal elements that were often celebrated in entrepreneurial culture (Keane and Chen, 2019). Wang explored the unique nature of cultural industries and how dynamics and evolution provided opportunities to expand four research themes of international business theory (internationalization strategy, cross-border innovation, social inclusion in the global economy, and emerging markets research). It aimed to provide a systematic review of current research, summarized the distinctive features of the cultural industry, and highlighted research gaps (Wang et al., 2020).

The goal of this research is to discuss the main factors that affect the competitiveness of the cultural industry and to construct an evaluation index system for the competitiveness of the cultural industry. The projective pursuit model and data envelopment analysis model based on the genetic algorithm (GA) are constructed by the selected evaluation method. The model is used to carry out an empirical study on the competitiveness level of the cultural industry. The influencing factors that restrict the competitiveness of the cultural industry in this region are analyzed. The reasons for lagging are found, and corresponding development measures are put forward according to the influencing factors.

Research on the development of cultural industry has always been a hot issue both domestically and internationally. For example, Duxbury revealed cultural and creative work in rural and remote areas through the study of cultural vitality, the rural creative class, the rural creative economy, and creative entrepreneurship. The results suggested an opportunity to fuse these discussions into a holistic approach to promoting cultural and creative work in rural and remote areas (Duxbury, 2021). Keane described the development of China’s creative cities from urban agglomerations to “characteristic towns,” reflecting the government’s desire to build characteristic cultural brands. There was an introduction to seeing digital technology as a solution to China’s social and economic problems and facilitating the realization of the “Chinese Dream” of national rejuvenation (Keane and Chen, 2019). Wang conducted a systematic review of current research, summarized the distinctive features of the cultural industry, and highlighted research gaps. How the unique nature, dynamics, and evolution of cultural industries provide opportunities to expand four research themes of international business theory were also illustrated (Wang et al., 2020).

The innovation is to find out the main factors that affect the competitiveness of the cultural industry and to construct its evaluation index system. The projective pursuit model and de model based on GA are constructed by the selected evaluation method. The model is used to perform an empirical study on the competitiveness level of the cultural industry. It explores the influencing factors that restrict the competitiveness of the cultural industry in this region and finds the reasons for its backward development. Then, the corresponding development measures are put forward according to the influencing factors.

The main structure of the study is as follows. Section “Introduction” firstly introduces the strategic background of rural revitalization. Section “Materials and methods” explains the difficulties faced by the cultural industry and its current development, and establishes an analytical model for the competitiveness of the cultural industry. Section “Results and discussion” explores the competitiveness level of the regional cultural industry. Through horizontal and vertical comparisons, it compares the average predicted values of the potential competitiveness systems in the five regions. Section “Conclusion” analyzes the numerical value of the experimental results and draws the research conclusion.

## Materials and methods

### Introduction to the cultural industry and its development dilemma

**FIGURE 1:**
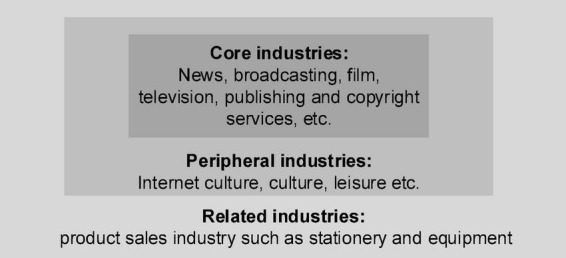
Cultural industry levels.

The understanding of the cultural industry here is under the regulations of the United Nations Educational, Scientific, and Cultural Organization. The cultural industry is defined as a series of activities that produce, reproduce, store, and distribute cultural products and services according to certain industrial standards (Xie et al., 2019). It is a kind of production and supply of spiritual products as the main activity to meet people’s cultural needs as the goal, namely the creation and sale of cultural significance. Cultural industries are classified according to the importance of their impact on society (Peukert, 2019), and their classification is illustrated in Figure 1.

In Figure 1, the cultural industry is divided into three layers, ten categories, and more than eighty subcategories (Yang et al., 2022). Cultural industries are social and economic, reflecting different social ideologies and market economy characteristics (Whitson et al., 2021). At present, the problems existing in the development of the Chinese cultural industry mainly include the following four points: (1) A complete and sound modern cultural market system has not been established, and the normal development of the cultural industry has been hindered. (2) The rich historical and cultural resources have not been fully developed. For example, the current film industry is still limited to the discovery of individual characters. It has not transformed China’s enviable cultural resource advantages into cultural industry advantages (Jiang et al., 2019). (3) The interests and functions of the management departments are seriously overlapping, which leads to the lag in the construction of cultural industry regulations. The cultural market management law has not yet been formed, and the development of cultural industries cannot be guaranteed at the legal level. (4) The service-oriented government is still under construction. There are problems such as poor systems and management in the current development of the cultural industry (Driskill and Rankin, 2020). Based on the above analysis, the cultural industry is a kind of enterprise cluster that can reflect the social ideology and the characteristics of the market economy and provide cultural products and services. There are still some development issues.

In the process of cultivating rural entrepreneurial culture, county governments, and village-level organizations should be committed to the comprehensive and coordinated development of agriculture and rural areas, promote the integrated development of rural primary, secondary, and tertiary industries, and steadily increase the income of rural residents while narrowing the gap between urban and rural residents’ living standards. To cultivate rural entrepreneurial culture and promote infrastructure construction, it is necessary to further promote the county and rural infrastructure construction and promote the development of rural cultural undertakings and industries under the spirit of improving the level of rural public services. The rural entrepreneurial culture has been cultivated, and the rural spiritual civilization and rural governance system have been further improved. High-quality talents have been attracted and introduced to help rural entrepreneurial activities and solidly improve the rural economy.

### Cultural industry competitiveness

The cultural industry has different industrial competitiveness from other industries. Industrial competitiveness refers to the competitive advantages of a country or region in the same industry in production efficiency, market share, and profitability. The competitiveness of the cultural industry is to obtain great output and profits with little input. It is necessary to continuously expand the market share of each market, and it is also important to allocate a reasonable product structure with limited resources (Feng, 2020). The competitiveness of cultural industries can be measured through the following five indicators: (1) The first is the abundance of cultural resources. It refers to a series of resources with humanistic values and traditional cultural values leftover from the process of human activities. (2) The second is the level of cultural environment. The demand for public infrastructure will lead to a unique scale and cluster effect on the cultural industry, which can improve the market allocation efficiency of cultural resources (Jiang and Luo, 2020). (3) The third is cultural production capacity. The rational and effective production of cultural products by cultural enterprises can solve the contradiction between the supply and demand of cultural products, enhance the competitiveness of the cultural industry, and promote the transformation and upgrading of the economic structure. (4) The fourth is the level of cultural consumption. A higher level of economic development generally leads to a higher level of consumption. It will promote the development of cultural products by cultural product manufacturers, and the product innovation ability will be strong. It is also beneficial for domestic cultural product manufacturers to expand their production scale and achieve economies of scale. (5) The fifth is government support. The government will issue different drainage measures due to the unique competitive advantages of the cultural industry such as high efficiency and no pollution. The government attracts cultural enterprises through tax relief and a relaxed environment for industrial development. However, if the government intervenes too much or does not act, the revolution of the cultural industry will be hindered during different periods of its development (Wardana et al., 2019).

Then there are related supporting industries. It has a close relationship with the competitiveness of the media industry and affects the competitiveness of the cultural industry. An industry that has no cooperation with other industries and companies will definitely not reach a strong level. If you have a strong ability, you should strive to be a leading industry and coordinate the development of other industries. If your ability is weaker, you must know how to depend on and learn from other strong industries. Through the division of labor and cooperation with other industries, industrial clusters are formed. In this industrial cluster effect, each link is reorganized and cooperated, which reduces the cost and investment risk of a single link, and improves the economic value of the industry as a whole. The development of the cultural industry can also learn from this model. The printing industry, media brokerage, and agency, publishing industry, education industry, etc. are all industries that can cooperate with each other, and gathering together can help build a complete cultural industry chain.

### Analysis model of cultural industry competitiveness

At present, the most well-known cultural industry competitiveness analysis model is the diamond model. The projection pursuit model is mainly used to construct its grade evaluation model. The BP-GA is to solve the optimal value of the projection pursuit index function. The following three models are reviewed and analyzed in turn.

**FIGURE 2:**
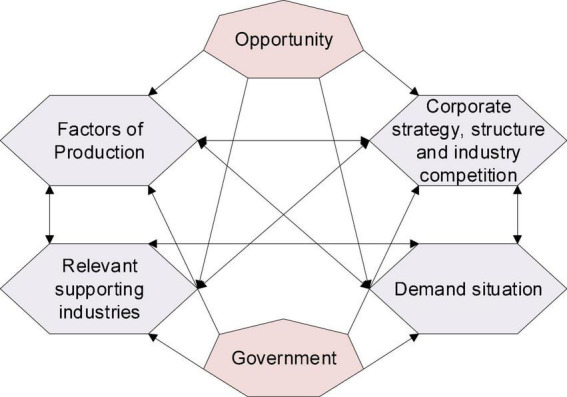
Porter’s diamond model structure.

(1) The first is the diamond model. The diamond model was first proposed by Michael E. Porter, a well-known strategic management scientist at Harvard Business School, to explain the magnitude of international competitiveness (Vlados, 2019). He believed that the international competitiveness of the industry depended on peers, substitutes, potential players, buyers, and suppliers. The basic competitiveness level of these factors affects the competitive advantage within the industry, and the difference in the role of these five factors leads to the difference in industry profitability (Yang, 2020). Figure 2 demonstrates the structure of the diamond model.

Figure 2 means that the four internal factors and two external factors that determine the competitiveness of the industry in the diamond model constitute a dynamic system that affects and restricts each other. The factors of production in the diamond model are the necessary foundations of the industry, including human resources, natural resources, knowledge resources, capital resources, and infrastructure (Erboz, 2020). Demand profile is the nature of domestic demand for an industry product or service. If domestic consumers are mature, sophisticated, and demanding, it will help the country’s companies gain an international competitive advantage. Because these features will force domestic companies to strive to achieve product high-quality standards and innovation (Chen et al., 2020). Relevant industries and supporting industries will be internationally competitive suppliers and related auxiliary industries and gradually extend the benefits of investment in advanced production factors to this industry. This helps the industry to gain a favorable position in international competition and assists the industry to gain competitive advantages (Jia et al., 2022). Enterprise strategy, structure, and competition refer to the domestic conditions governing the creation, organization, and management of firms and the nature of domestic competition. Different countries have different “management ideologies” with different characteristics. These “management ideologies” help or hinder the development of a country’s competitive advantage. There is a link between the presence of intense domestic competition within an industry and its ability to maintain a competitive advantage. The government can provide the resources needed by enterprises, create an environment for industrial development, and bring new opportunities and pressures. The government’s direct investment should be in areas in which enterprises cannot act, such as developing infrastructure, opening up capital channels, and cultivating information integration capabilities. The government indirectly affects the competitiveness of the industry by directly affecting the above four internal factors. Seizing the opportunity is related to the life and death of an enterprise. Opportunities often go both ways. The new competitor gains an advantage, and the old competitor loses the advantage. Manufacturers that can meet new demands will seize the “opportunity” (Liu et al., 2018; Liu Y. et al., 2020).

**FIGURE 3:**
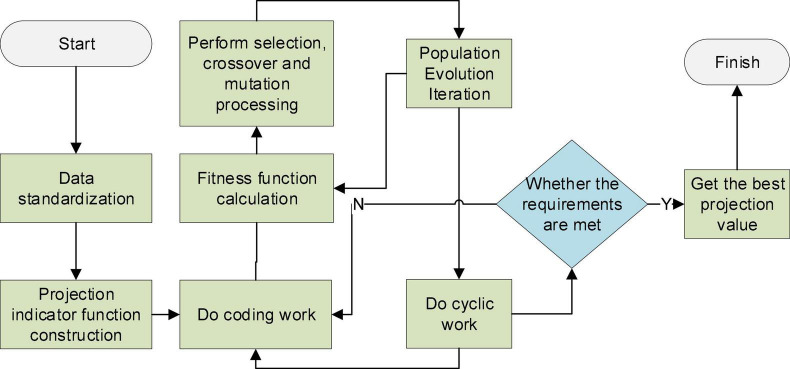
Flow chart of the projection pursuit (PP) model based on genetic algorithm (GA).

(2) The second is the projection pursuit (PP) model based on the GA. The PP model is an exploratory data analysis method directly driven by samples (Shao et al., 2021). The basic principle is to project the high-dimensional data structure or feature to be studied onto the low-dimensional subspace in some way. The projected configuration is described by the projected index function. Then, the possibility of showing a certain classification and ranking structure of the original system during the projection process is obtained, and the projection value of the optimal projection index function is found from it (Yu et al., 2020). The GA was proposed by John Holland in the 1970s by the evolutionary laws of organisms in nature. It is a computational model of the biological evolution process that simulates the natural selection and genetic mechanism in Darwin’s theory of biological evolution. It can solve the optimal solution of the objective function in the mathematical model. GA has been widely used in modern high-tech intelligence fields such as combinatorial optimization, machine learning, signal processing, and artificial life (ZainEldin et al., 2020). The GA is used to solve the optimal solution of the objective function to solve the optimal value of the projection index function of PP. Its model flow chart is shown in Figure 3.

In Figure 3, the optimal solution to some high-dimensional, non-normal, and non-linear complex problems can be studied by combining the two methods. This principle can be used to implement an evaluation model of the competitiveness level of the cultural industry. The model cannot only rank the samples but also feedback on the influence of the indicators on the results through the projection direction (Memon et al., 2021). The four steps in implementing the model are the following:

(1) The index data is normalized first. The connotation represented by each indicator is different, so it is necessary to perform dimensionless processing or standardization processing on the indicator data.


(1) $$x\,\left(i,j\right)=\left[x^{*}\,\left(i,j\right)-x_{m\,i\,n}\,\left(i,j\right)\right]/\left[x_{m\,a\,x}\,\left(i,j\right)-x_{m\,i\,n}\,\left(i,j\right)\right]$$

In Equation (1), x* (i,j) represents the *j*th index value for the *i*th sample. *x*_*max*_ (i,j) and *x*_*min*_ (i,j) are the maximum and minimum values of the *j*th indicator, respectively.

(2) Projection pursuit can process the one-dimensional projection value *z*(*i*) of P-dimensional data {*x*(*i*, *j*) | *j* = 1∼ *p*} in the projection direction of *a* = [*a*(1), *a*(1), …, *a*(*p*)].


(2) $$z\,\left(i\right)=\sum_{j=1}^{p}a\,\left(j\right)\,x\,\left(i,j\right)$$

In Equation (2), *a* is a unit-length vector. The constructed projection index function is to compress the local projection points scattered by the projection value *z*(*i*).


(3) $$Q\,\left(a\right)=S_{Z}\,D_{Z}$$

In Equation (3), *S*_*Z*_ expresses the standard deviation of the projected values, and *D*_*Z*_ shows the local density of the projected values *z*(*i*).


(4) $$S_{z}={\left[\sum_{i=1}^{n}{\left(z\,\left(i\right)-E\,z\right)}^{2}/\left(n-1\right)\right]}^{0.5}$$

(5) $$D_{z}=\sum_{i=1}^{n}\sum_{j=1}^{n}\left(R-r_{i\,j}\right)\,u\,\left(R-r_{i\,j}\right)$$

In Equations (4, 5), *E*_*z*_ means the mean of *z*(*i*). *R* indicates the density window width, which is generally 0.1*S*_*z*_. r_*ij*_ is the distance between samples. *u*() refers to the corresponding leap function.

(3) The projection index function needs to be optimized. When the sample set of each index value does not change, the projection index function *Q*(*a*) will only change with the projection direction, and its optimal projection direction is as follows:


$$m\,a\,x\,Q\,\left(a\right)=S_{Z}\,D_{Z}$$

(6) $$s.t.\sum_{j=1}^{p}a^{2}\,\left(j\right)=1$$

*Q*(*a*) is used as the objective function, and the projection *a*(*j*) of each indicator is the optimization scalar. The optimal projection direction a*(*j*) can be obtained by the GA. Then, the *z*(*i*) value of each sample is calculated, and the comprehensive evaluation is carried out by sorting the *z*(*i*) value.

(4) A clustering model is established. The obtained a*(*j*) is substituted into Equation (2) to acquire the projection value z*(*i*) of each sample. In addition, the sample set of each index can be classified and analyzed by sorting according to the projection value z*(*i*).

The assumption made here is: Industrial culture based on entrepreneurship significantly shapes the ideology of management from which the key factors of macro-competitiveness develop. The first step in conducting empirical research is to collect data on the level of competitiveness of cultural industries in a certain region and compared regions. Here, panel data from eleven regions are selected for analysis. The data come from statistical data, such as “Development Index of Cultural Industries in China’s Provinces and Cities,” “Statistical Yearbook of Chinese Culture and Related Industries,” “Statistical Yearbook of China’s Regional Economy,” and “Statistical Bulletin of Regional Development.”

## Results and discussion

### Experimental dataset

The first step in empirical research is to collect data on the competitiveness of cultural industries in a region and the region to be compared. It selects panel data from 11 regions for analysis. The data are all from the official website of the National Bureau of Statistics and documents such as “Development Index of Cultural Industries in China’s Provinces and Cities,” “Statistical Yearbook of Chinese Culture and Related Industries,” “Statistical Yearbook of China’s Regional Economy,” “Statistical Bulletin of Regional Development,” and other documents.

### Parameter setting

The collected data and data are sorted. The Kaiser-Meyer-Olkin (KMO) statistic is used to determine the overall correlation degree of the variables based on the simple correlation and partial correlation coefficient between each variable. The correlation is strong, the partial correlation coefficient is much smaller than the simple correlation coefficient, and the KMO value is close to 1. In general, if KMO > 0.9, it is very suitable for factor analysis; if 0.8 < KMO < 0.9, it is suitable; if it is above 0.7, it is more suitable to use the KMO test for analysis. The SPSS software is used for data processing, the software running system adopts Microsoft Windows XP or 2000, the minimum available drive space is 400 MB, the memory size is 256 MB, and the load of the Central Processing Unit (CPU) is 1.5. By analyzing the experimental data, the development trend of the entrepreneurial cultural industry background in the context of the rural revitalization strategy can be drawn.

### Cultural industry in the region

**FIGURE 4:**
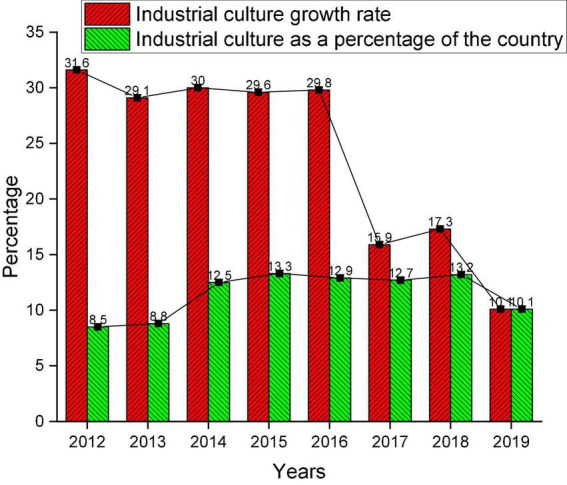
The situation of the cultural industry in the region in the past 8 years.

According to the data from the “Development Index of Cultural Industries in China’s Provinces and Cities,” the situation of the cultural industry in the study region in the past 8 years is demonstrated in Figure 4.

Figure 4 indicates that the development of cultural industries in the region is optimistic. Many indexes of the cultural industry are at the forefront of the country. It is worth noting that the productivity index, influence index, and comprehensive index are all good. It is concluded that the cultural industry has become a new growth point in the region’s economy.

### Vertical analysis and evaluation of the competitiveness level of cultural industry in the region

**FIGURE 5:**
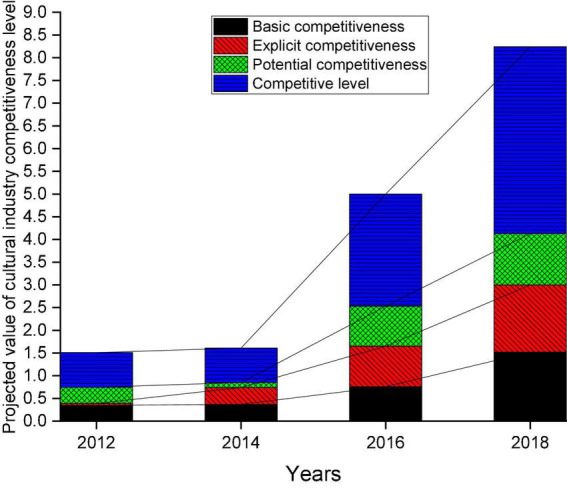
Competitiveness level and trend of the projected value of each subsystem.

The competitiveness level of the region and the projected value trend of each subsystem are analyzed. The results are shown in Figure 5.

**FIGURE 6:**
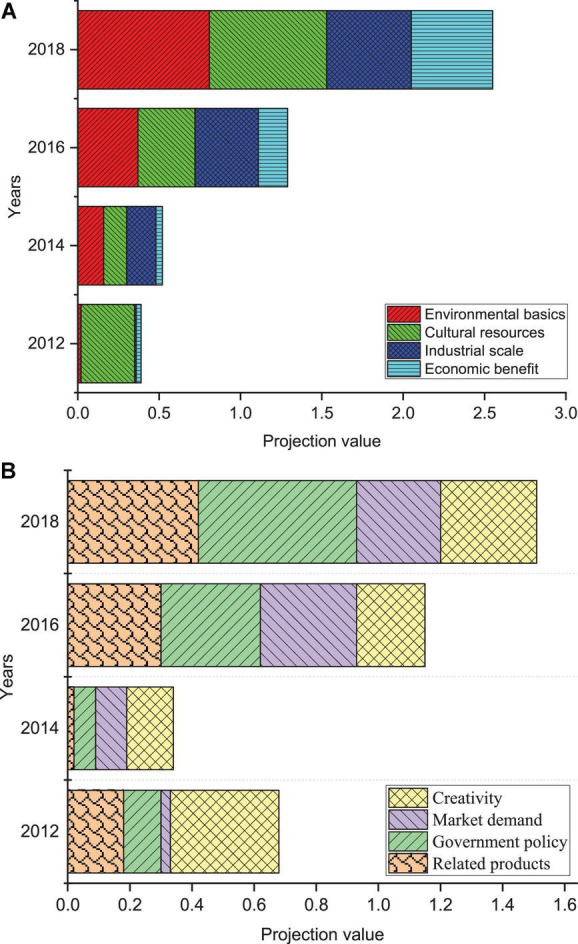
Trend of projection value of criterion layer of industrial culture subsystem. **(A)** Trends in projected values of environmental foundation, cultural resources, industrial scale, and economic benefits. **(B)** Projected value trends of related industries, government policies, market demands, and innovations.

In Figure 5, on the whole, the projected value of its cultural industry competitiveness level is increasing every year. Especially after 2014, the level of competitiveness has improved rapidly. From the perspective of the projected values of each subsystem, the apparent competitiveness of the three subsystems has increased significantly, but the relative growth rate of potential competitiveness has slowed down. From the perspective of subsystems, the relatively flat level of competitiveness has led to slow growth in previous years. The projection value of the criterion layer under each subsystem can be displayed in detail to deeply analyze the internal change trend of the competitiveness system in this region. The calculation results are revealed in Figure 6.

In Figure 6, it is concluded that the internal change trend of the competitiveness system in this region has been significantly improved in the eight criterion layers as a whole from the trend change of the projected value of the industrial culture subsystem criterion layer. All eight criterion layers positively impact the competitiveness level projected value during this period. The basic competitiveness in industrial culture includes environmental foundation and cultural resources. The projected score for the environmental base of the region has grown over time. However, cultural resources decline in 2014, indicating that the mining of cultural resources is not paid much attention this year. Furthermore, the growth rate of two criterion layers in the basic competitiveness system of the region far exceeds that of other criterion layers. The dominant competitiveness in industrial culture has three standard layers: industrial scale, economic benefit, and related industries. The scale of the industry has grown year by year, but the growth rate will slow down after the scale reaches a certain level. The main reason is that financing problems restrict the further expansion of the industrial scale in the process of industrial transformation. The economic benefits are still increasing during this period, corresponding to changes in the scale of the industry. When the industrial scale gradually expands, the economic benefits increase slowly. The projected value of related industries also rises with the overall economic development of the region over time.

From the horizontal comparison of the three standard layers of industrial scale, economic benefits, and related industries, the total amount of related industries is small. The region needs to expand the field of cultural industry from this aspect. The potential competitiveness in industrial culture includes three standard layers: government policy, market demand, and innovation ability. In the part of government policy projection, the score is low in 2014 due to less related cultural policies and weak financial support. For the trend of scores in market demand, its changes are ups and downs, which are affected by the international situation and regional personnel and cultural expenditures. The ability to innovate has been fluctuating slowly, indicating that the region has not paid enough attention to this standard level. In a word, the market demand and innovation ability are the weak links of the cultural industry in this region, and the government must focus on development.

### Horizontal analysis and evaluation of the competitiveness level of cultural industries in the region

Although the studied region cannot be compared horizontally with the projected values of other regions, the average value of each year’s data can be used to reflect the evaluation of the competitiveness level of the cultural and creative industries in each region during the study period.

**FIGURE 7:**
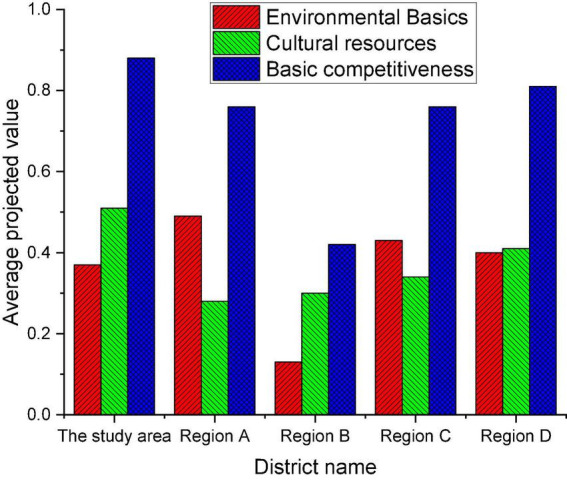
The average projected value of the basic competitiveness system in five regions.

(1) The average projected value of the basic competitiveness system in each region is shown in Figure 7.

Figure 7 denotes that the projected value of the basic competitiveness system for the five regions all exceeds 0.4. Among them, the basic competitiveness of the studied region is 0.8, ranking first and showing strong basic competitiveness. Moreover, cultural resources are also the most advantageous projects from the standard level, which also makes the projected value of basic competitiveness high. From the point of the environmental foundation, the score is not outstanding, illustrating that the investment in environment-related infrastructure construction needs to be improved. The industrial competitiveness of other regions shows an obvious internal imbalance, which deserves attention.

**FIGURE 8:**
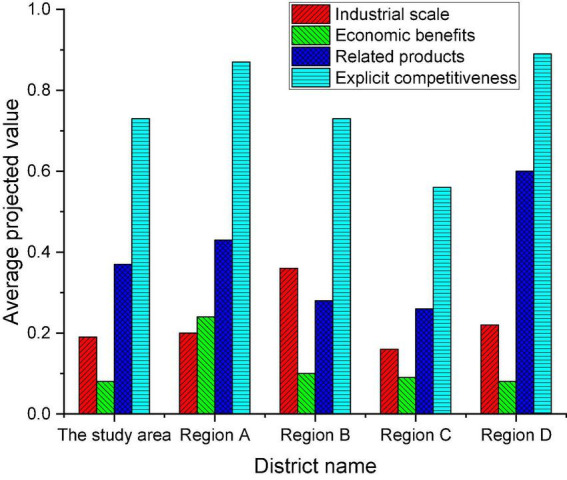
The average projected value of the dominant competitiveness system in five regions.

(2) Figure 8 shows the systematic analysis of the dominant competitiveness of each region.

**FIGURE 9:**
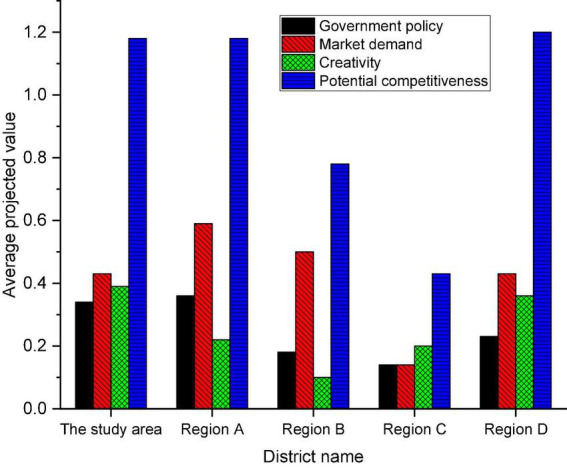
The average projected value of the potential competitiveness system in five regions.

Figure 8 demonstrates that all five regions exceed 0.5 on the average projected value of dominant competitiveness. The average projected value of dominant competitiveness in the region has exceeded 0.7, which is in the middle of the five regions. The results imply that there are differences in the degree of economic development in different regions, but overall they are excellent. In detail, there is little difference between regions in the criterion layer of the industrial scale of the dominant competitiveness system. The studied regions are in the middle of the economic development level, and the scale ranks in the middle. China’s cultural and creative industry starts late, and there is still great potential for development and overall scale. The level difference of related industries in the three standard layers has the most obvious change, and the changing trend is almost consistent with the overall change of dominant competitiveness. When the size of the economy is larger, the development performance of related industries is better, indicating an obvious positive correlation. From the analysis of economic benefits, the benefits of cultural and creative industries greatly facilitate economic development. The region still needs to improve industrial competitiveness by promoting economic efficiency. Finally, there is a systematic analysis of the potential competitiveness of this region and the rest of the region, and the results are revealed in Figure 9.

Figure 9 manifests that the potential competitiveness of the region exceeds 1.1. This shows that the cultural industry has great potential for development, and a large cultural industry will positively promote the overall competitiveness of the industry. The projected value of Region A is the highest in terms of government policy from the perspective of the three criterion layers of potential competitiveness: government policy, market demand, and innovation capability. This indicates that it attaches great importance to the cultural industry, and the government policy studied here needs to continue to be strengthened.

At the level of market demand, the region still needs to continue to improve the market system and stimulate public consumption. Although innovation capability has a high score in this region, innovation capability has always been the most important competitiveness for the potential competitiveness of the cultural industry. Therefore, attention should be paid to the core innovation ability in entrepreneurship to facilitate the development of the cultural industry.

The rural revitalization strategy has advantages for the entire cultural industry. On the one hand, it can free many people from the troubles of basic living, and people will naturally increase their consumption of cultural industries. On the other hand, it can also inject new vitality into the status quo of the cultural industry, and the existing culture and rural culture will further collide and integrate. Especially under the severe impact of the current COVID-19, the gradual sinking of the cultural industry into the rural market is also a very valuable attempt.

## Conclusion

The competitiveness of a region’s cultural industry is studied through Porter’s diamond model. The regional cultural industry situation is discussed first. Then, the vertical and horizontal analyses of the competitiveness level of the cultural industry are performed. Next, the differences in the base, dominant, and potential competitiveness of cultural industries in this region with other regions are compared. The influencing factors that restrict the competitiveness of the cultural industry in the region are analyzed. At last, the reasons for its backward development are obtained, and corresponding development measures are put forward according to the influencing factors. There are still many problems left unresolved due to research time and personal ability. For example, problems such as how to form a regional industrial enterprise group and how to use the regional characteristic culture to create a cultural industry chain have not been resolved. These issues will be studied in the future to contribute to the development of the Chinese cultural industry.

From the above results, the previous hypothesis has been fully confirmed. The competitiveness of cultural industries in a certain region is researched through the Porter diamond model. The cultural industry in the region and the level of competitiveness of the cultural industry are vertically and horizontally analyzed. The foundation and apparent potential competitiveness of the cultural industry in the region are identified, and where its level is located in other regions is determined. Then, the influencing factors that restrict the competitiveness of the cultural industry are analyzed, and the reasons for its backward development are found. Besides, the corresponding development measures are proposed according to the influencing factors. In the process of the continuous realization of the rural revitalization strategy, if the cultural industry can follow the trend, it will not only add luster to the revitalization strategy but also allow it to find further opportunities and develop its potential. Compared with the previous theoretical research, the evaluation index system of cultural industry competitiveness is constructed. Conclusions are drawn through data analysis, with scientific and research potential. However, there are still many problems that have not been solved due to factors such as research time and personal ability, such as how to form a regional industrial enterprise group and how to use the regional characteristic culture to create a cultural industry chain. In the future, such issues will be studied to contribute to the Chinese cultural industry.

## Data availability statement

The raw data supporting the conclusions of this article will be made available by the authors, without undue reservation.

## Ethics statement

The studies involving human participants were reviewed and approved by Ocean University of China Ethics Committee. The patients/participants provided their written informed consent to participate in this study. Written informed consent was obtained from the individual(s) for the publication of any potentially identifiable images or data included in this article.

## Author contributions

The author confirms being the sole contributor of this work and has approved it for publication.
